# 

**Published:** 1918-12-07

**Authors:** 


### THE COLLEGE OF NURSING

**(Limited by Guarantee).**

### EDINBURGH CENTRE.

THE third meeting of the Centre under the auspices of the National Council for Combating Venereal Diseases was held on November 26. Miss Turnbull, R.R.C., Matron of the Deaconess Hospital, in welcoming the lecturer, Dr. Mary Macnicol, said she was delighted, as vice-chairman of the Scottish Midwives' Association, to have the opportunity of saying how much she had appreciated the presence of so many midwives at meetings where they would learn so much of vital importance in their work. Dr. Mary Macnicol, who received a most cordial reception from her audience, dealt briefly but clearly with the symptoms and nature of venereal diseases, and explained the various stages of syphilis and the importance of treatment at an early stage. She emphasised the way in which gonorrhœa was easily spread by use of towels, etc., used by an infected person, and stated how these diseases were contagious rather than infectious.

### LONDON CENTRE: ST. THOMAS'S HOSPITAL, S.E. 1.

(Hon. Secretary, Miss M. M. BIGGAR.)

*Thursday, December 12, 7* P.M.—A meeting convened by the London Centre of the College of Nursing will be held at the Hospital for Consumption and Diseases of the Chest, Brompton, S.W. The subject to be discussed is "The Position of Nurses Being Trained in Small and Special Hospitals in Reference to Registration." Matrons of these hospitals have been invited to attend so that expert opinion may be obtained on this important subject.

### Matrons' and Sisters' Appointments.

BIDEFORD AND DISTRICT HOSPITAL.—Miss Jennie Jones has been appointed matron. She was trained at the Llanelly General and Eye Hospital, has held posts at that hospital, the Cancer Hospital, London, S.W., Aberystwyth Infirmary, and the Cardigan General Hospital.

CHILDREN'S HOSPITAL, SHEFFIELD.—Miss May Stanley has been appointed sister. She was trained at the Swansea General and Eye Hospital, where she was later temporary sister.

COTTAGE HOSPITAL, ELTHAM.—Miss Mary Young Thomson has been appointed matron. She was trained at Addenbrooke's Hospital, Cambridge; has been theatre sister, women's surgical-ward sister and assistant matron at Worcester General Infirmary; sister under Queen Alexandra's Imperial Military Nursing Service Reserve at the Royal Herbert Hospital, Woolwich, and in the Salonika Field Force. Miss Thomson holds the certificate of the Central Midwives Board and is a member of the College of Nursing.

GLASGOW ROYAL ASYLUM, GARTNAVEL.—Sisters Florence M. Gordon Duff and Eva Cuthbert have been appointed assistant matrons. Sister Gordon Duff was trained at Epsom Infirmary, and has since been doing private nursing. Sister Cuthbert was trained at Belfast Infirmary.

INFECTIOUS DISEASES HOSPITAL, TAMWORTH.—Miss Mary Borton has been appointed matron. She was trained at the Oakwell Joint Hospital, Birstall, Leeds, and at the West Bromwich and District Hospital. She has since been charge nurse at Stanfield Hospital, Burslem, sister, night superintendent, and deputy matron at Stafford Borough Isolation Hospital.

ISOLATION HOSPITAL, MALVERN.—Miss J. E. Gilbert has been appointed matron. She was trained at the Royal Berkshire Hospital, and at the City Hospital, Edinburgh; has been ward sister and deputy matron at the Infectious Diseases Hospital, Huddersfield; sister-in-charge of the Sankey Sanatorium, Warrington; and superintendent of nurses at the Fever Hospital, Blackburn.

JESSOP HOSPITAL FOR WOMEN, SHEFFIELD.—Miss Dora Wilkinson has been appointed ward sister. She was trained at Preston Royal Infirmary, and has also taken sister's duties there.

SAMARITAN FREE HOSPITAL.—Miss Winifred Tice has been appointed matron. She was trained at St. Bartholomew's Hospital, and has been sister-in-charge under Queen Alexandra's Imperial Military Nursing Service Reserve at a clearing-station in France, and at the Military Hospital, Colchester.

THE INFIRMARY, BRIDGWATER.—Miss S. A. Lewis has been appointed head nurse. She was trained at Crumpsall Infirmary, Manchester, and has since held posts under Wolverhampton, Barnsley, Congleton, Bishop's Stortford, Brighton, East Preston, and Plymouth Unions.

**NOTE.—Particulars of Appointments for Insertion in this column will be welcomed.**

